# Tools of discovery

**DOI:** 10.1002/msb.201305048

**Published:** 2014-01-09

**Authors:** Thomas Lemberger

**Affiliations:** ^1^ EMBOHeidelbergGermany

## Abstract

*Molecular Systems Biology* has transitioned to the new *EMBO Press* publishing platform to offer a richer and more transparent access to high‐quality research.

When *Molecular Systems Biology* was launched in 2005 as an Open Access journal, the scientific motivation was to disseminate immediately the results produced by the systems biology community and to make them globally available across all the life sciences. Sharing, analyzing and integrating data are central to systems biology. As George Church noted in his inaugural Editorial, “It is an important exercise for each of us, as we contemplate publishing our latest discovery to ask how we can make this more accessible for checking and merging with other discoveries” (Church, 2005). *Molecular Systems Biology* has now relaunched on the new *EMBO Press* platform to further develop this vision of a progressive and open journal that catalyzes discovery.

### Editorial continuity and transparency

The Editorial Team of *Molecular Systems Biology* at EMBO has worked hard with the journal's Senior Editors and its Advisory Editorial Board to ensure the high quality of the science published in the journal and this will remain our priority. *EMBO Press* now provides a new publishing environment that will allow *Molecular Systems Biology* to continue to develop as an Open Access journal that sets a higher priority on scientific quality and editorial independence than on financial targets.

The journal remains devoted to the fields of systems biology, synthetic biology and systems medicine and the breadth of its scope includes all flavours of systems biology—from the mechanistic quantitative analysis of the dynamics of biological circuits to the understanding of complex diseases by the integration of genome‐wide datasets. New fields continue to burgeon and we are very excited by the new frontiers emerging from the latest advances in single‐cell and single‐molecule *in vivo* biochemistry, whole‐cell modeling, genome‐scale engineering and personalized systems genetics.

Selecting for quality requires critical evaluation and thorough peer review. To make this process less arcane and expose the inner working of the journal, three years ago we implemented a transparent editorial process (Lemberger, 2010) whereby anonymous referee reports and editorial decisions are published alongside our papers. We now circulate the referee reports among the reviewers before making an editorial decision and this provides the referees with the opportunity to discuss each others’ evaluation and to catch potential ‘outlier’ recommendations. These sets of policies for more transparency and fairness are the core of the *EMBO Press* editorial philosophy.

### Multidimensional papers

The new online platform provides direct access to the content of the papers and removes the clutter that distracts readers from the science. The interface is based on the concept of a multidimensional paper that offers access to the content at various levels of detail – general readers need quick access to summaries to browse through the content whereas experts will reanalyze the data and sometimes will want to re‐use them (Fig 1). By providing textual and visual synopses, by replacing the classical supplementary information with integrated ‘Expanded View’ sections, and by providing easy access to reusable datasets and software code, the new interface will provide readers with intuitive tools to choose the level of depth that matches their needs.

**Figure 1:**
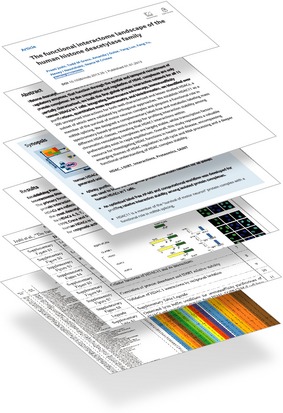
The multidimensional paper A scientific article can be envisioned as juxtaposed layers—Title, Abstract, Synopsis, Article, Expanded View and Datasets—that provide access to the paper with increasing resolution and allow readers to zoom in or out to access the information at the required level of granularity.

For papers accepted from 1^st^ January 2014, we will replace the traditional ‘supplementary information’ section—in our view, an outdated legacy of the print era—by ‘Expanded View’ sections. Expanded Views will be integrated in the flow of the paper and include either visually rendered objects (figures, small tables, text) or downloadable files for structured data, models and computer code. To avoid such sections becoming an undefined ‘dumping ground’ for data that are only peripherally related to the main study, their scope will be defined to only include information that is directly related to the scientific arguments of the paper, including calibration, optimization and control data, benchmarking analysis, negative results, detailed algorithms, mathematical demonstrations or complex protocols (http://msb.embopress.org/authorguide). While the general reader may elect to collapse such detailed ‘Expanded View’ sections, they contain important data for the expert reader and deserve to be displayed and reviewed as an integral part of a paper.

### Access to data

Experimental data and computational models are the foundational layer of work published in *Molecular Systems Biology* (Fig 1). Improving access to this core component of the paper will be one of the priorities for the development of the *EMBO Press* platform. *Molecular Systems Biology* will continue to request at submission that datasets and computational models are deposited in public repositories whenever applicable and to host datasets and computer code with the paper when no such public resource exists. In addition, as for the last four years, *Molecular Systems Biology* encourages authors to upload ‘source data’ files that contain the actual data points and measurements depicted in figures so that readers can download and reanalyse them (Lemberger, 2010). This is particularly relevant for quantitative data used to calibrate or validate systems biology models, but it is also important to show images of blots or microscopic images in full without the need to fit them by cropping within the narrow space allocated to conventional figure panels.

While Open Access has been instrumental in facilitating global literature mining, mainly through text‐based methods, it is crucial to extend access to published data and models. To lower potential legal barriers to text mining and data reuse, all papers published in *Molecular Systems Biology* will be published under a Creative Commons licence (http://creativecommons.org/licenses/by/4.0/‎) and the datasets and source data hosted by the journal will be released under the Creative Commons waiver CC0 (http://creativecommons.org/publicdomain/zero/1.0/). Naturally, removing such legal hurdles will not diminish in any manner the need to respect the standard scholarly practice of citing the source whenever possible and to provide appropriate credit when using the work of others.

### Biological metadata are gold

Data result from carefully designed experiments and their structure reflect the experimental design. Metadata that describe the biological content of research data and their structure are a gold mine of information that is usually only available as narrative descriptions in the text of an article. To enable new data‐oriented search strategies and improve the machine readability of the data integrated in published papers, *EMBO Press* will develop the *SourceData* platform to encode machine‐readable biological metadata associated with figures and their source data. It will also establish internal curation and semantic enrichment steps with data editors who are embedded in the production process.

It is not the remit of journals to substitute for domain‐specific biological databases. The comprehensive in‐depth curation performed by public resources such as *UniprotKB/Swiss‐Prot*, for example, remains invaluable. There are however still many data types, in particular for small‐scale assays, for which there are no dedicated structured databases, and new assays emerge all the time. Journals still invest much effort to copy editing and typesetting activities whereas little time is spent on the data that are at the core of a scientific article. By shifting the balance towards devoting more attention to the data published in our journals, we wish to bridge the gap between journals and biological database and open the door to new strategies to search, mine and compare the data published in the scientific literature for the benefit of the scientific community.

### Feedback loop

Many challenges lie ahead. One is the rigorous and complete description of experimental and computational methods. Together with access to data and metadata, traceable methodological procedures are essential to ensure the reproducibility of the scientific record and we welcome suggestions from our readers on how this can be achieved (contact msb@embo.org). We are excited at the prospect of collaborating with our publishing partners *Wiley‐Blackwell* and *HighWire Press* to develop the *EMBO Press* platform as a flexible infrastructure. With *EMBO Press* and its four journals, our vision is to transform, step by step, the traditional scientific article into a community resource that can be used by researchers as a powerful tool of discovery.
